# Transverse Rupture of Segment II (Couinaud) of the Left Hepatic Lobe in Deceleration Trauma: Morphological Characteristics and a Strategy for Intraoperative Detection

**DOI:** 10.3390/jcm14144889

**Published:** 2025-07-10

**Authors:** Piotr Arkuszewski, Zbigniew Pasieka, Jacek Śmigielski, Karol Kłosiński

**Affiliations:** 1 Department of Biomedicine and Experimental Surgery, Faculty of Medicine, Medical University of Lodz, 90-136 Lodz, Poland; zbigniew.pasieka@umed.lodz.pl; 2 Department of General and Oncological Surgery, Primate Cardinal Stefan Wyszyński Provincial Hospital in Sieradz, 98-200 Sieradz, Poland; smiglo@mp.pl

**Keywords:** blunt trauma, deceleration, rupture, liver, left lobe, hepatic ligaments, intraoperative assessment

## Abstract

**Background/Objectives:** Deceleration can cause liver ruptures via ligament traction, with a specific, little-known transverse rupture in segment II of the left lobe being a concern. This study aimed to provide a detailed morphological characterization of these segment II ruptures, analyse their formation mechanisms using autopsy material, and propose a systematic intraoperative assessment method to improve their detection. **Methods:** This study analysed the autopsy cases of 132 victims of sudden, violent deceleration (falls from height, traffic accidents) performed between 2011 and 2014. Liver injuries were meticulously described, focusing on the morphological characteristics of ruptures (course, shape, depth) and their location relative to hepatic ligaments. Cases with prior liver resection due to injuries were excluded. **Results:** Liver ruptures were found in 61 of the 132 analysed cases (46.2%). A “new location” for ruptures was identified on the diaphragmatic surface of the left lobe’s segment II, near and along the left coronary and triangular ligaments. This specific type of rupture was found in 14 cases. Overall, 40 cadavers had liver ruptures near ligaments, totalling 55 such distinct ruptures, indicating that some had multiple ligament-associated tears. The incidence of liver rupture at this newly described site was statistically significant. **Conclusions:** Transverse rupture of the left hepatic lobe’s segment II, in its subdiaphragmatic area, results from ligament “pulling” forces during deceleration and is a characteristic injury. Its presence should be considered following blunt abdominal trauma involving deceleration, and the subdiaphragmatic area of the left lateral lobe requires intraoperative inspection, especially if other ligament-associated liver ruptures are found.

## 1. Introduction

Blunt liver injuries are, together with penetrating injuries, the main types of post-traumatic injury to this organ. They represent one of the most serious problems in trauma surgery. Their mechanisms are complex and depend on the vector and magnitude of the acting force. The formation of liver ruptures due to the tearing action of ligaments caused by deceleration is a well-known and well-described mechanism of blunt abdominal trauma leading to injury in the professional literature [1,2,3,4]. The mechanism is quite complex, with the main consecutive components being listed as follows:

- Rapid displacement of the body as a result of inertial forces;
- Further movement of the liver as a result of inertia forces after the body has come to a standstill due to, for example, hitting the steering wheel or dashboard (in traffic accidents) or hitting the ground (in falls from heights);
- Stretching of the hepatic ligaments following movement of the liver;
- Pulling and straining of the liver parenchyma leading to shearing forces;
- Further stretching of the liver ligaments and pulling of the liver parenchyma, resulting in rupture of the capsule and parenchyma of the organ at the sites of attachment to the diaphragm and abdominal wall [1,2,3,4].

The characteristic sites of hepatic ruptures as described above in relation to the Couinaud segmentation of the liver are listed as follows:

- The diaphragmatic surface of the right lobe between both sectors, in the plane between Couinaud segments VI/VII and V/VIII, with the tear parallel to the right triangular hepatic ligament;
- The left lobe to the right (in segment IV) or to the left (in segment III) of the falciform ligament with a tear located next to and parallel to this ligament [1,2,5,6,7].

These injuries are not difficult to see during a laparotomy. This is because the ruptures are longitudinal, parallel to the long axis of the body, and are not located in the upper part of the organ. However, a transverse rupture of the left hepatic lobe in the subdiaphragmatic area, within segment II according to Couinaud, running parallel to the left coronary ligament and the left triangular ligament, has occasionally been noted in the professional literature [8,9]. Due to its features and location, it is not readily discernible on simple, even visual, assessment of the liver and may go unnoticed or even overlooked during laparotomy. The potential serious consequences of overlooking this type of injury, such as the formation of a haematoma or biloma, represent a significant clinical challenge. Given the above, the aim of the present study is to provide a detailed morphological characterization of transverse segment II ruptures of the left hepatic lobe arising in a deceleration mechanism. An additional aim was to analyse the mechanisms of their formation on the basis of autopsy material and, crucially from a clinical perspective, to propose a systematic method for intraoperative assessment of the liver aimed at increasing the detection of this “insidious” injury.

## 2. Materials and Methods

The research material consisted of autopsy cases performed at the Department of Forensic Medicine, Medical University of Lodz, between 1 September 2011 and 15 April 2014, concerning deaths in circumstances in which there was a sudden, violent deceleration of the body and deceleration was triggered. These were the following post-mortem cases:

- Victims of falls from height (even relatively low, e.g., from the first floor, but from a height other than their own standing height);
- Victims of traffic accidents, but only drivers and passengers of a car that collided with another car or a fixed obstacle, e.g., a tree).

Cases of deaths occurring both at the scene and later in hospital were analysed. The study was prospective in nature.

The main criterion for exclusion from the study was resection of the injured area of the liver.

The study received a positive and unqualified opinion from the Bioethics Committee at the Medical University of Lodz for further use (Resolution of the Bioethics Committee on the design of the medical experiment No. RNN/205/14/KE). All data used in the study were fully anonymized to protect the privacy of the deceased. The study and its results formed the starting point for the doctoral dissertation entitled “OCENA MECHANIZMÓW POWSTAWANIA PĘKNIĘĆ WĄTROBY W URAZACH DECELERACYJNYCH ZE SZCZEGÓLNYM UWZGLĘDNIENIEM ROLI WIĘZADEŁ WĄTROBY” (“EVALUATION OF THE MECHANISMS OF LIVER RUPTURE IN DECELERATION INJURIES WITH PARTICULAR REFERENCE TO THE ROLE OF HEPATIC LIGAMENTS”), successfully defended at the Medical University of Lodz in 2015.

The investigations were carried out as part of the routine post-mortem diagnostics performed during autopsies. However, the examination was extended to include very detailed descriptions of liver injuries, taking into account the following morphological characteristics of ruptures of this organ:

- Course and shape (longitudinal, transverse, stellate);
- Depth (capsular damage, superficial parenchymal damage, deep parenchymal damage, full-thickness rupture of the organ).

In addition, the number of ruptures and their location within one of the lobes and in relation to other characteristic liver structures (primarily ligaments) were described. The liver was assessed visually in situ and in tabula—both before and after dissection—when it was possible to assess areas of the liver located extra-circularly (i.e., not on the liver surface or directly under the hepatic capsule).

## 3. Results

The group of fatal victims of falls from height comprised 47 cases. The group of fatal victims of traffic accidents numbered 85 cases. In total, the group of people who had been exposed to deceleration injury just before death thus numbered 132 cases. Falls from height accounted for 35.6% (47/132) and traffic accidents for 64.4% (85/132). A total of 25 cases of liver rupture were related to falls from height, accounting for 18.9% of all cases (25/132), and 36 were related to traffic accidents, accounting for 27.3% of all deaths analysed (36/132). Liver rupture was found in a total of 61 cases out of 132 analysed, representing 46.2%. Of these 61 cases, 41.0% were liver ruptures following falls from height (25/61), and 59.0% (36/61) were liver ruptures following traffic accidents. Liver rupture was more common in falls from heights (25/47—53.2%) than in motor vehicle accidents (36/85—42.35%).

The initial assessment already showed that liver ruptures were found in a “new location”, i.e., on the diaphragmatic surface of the left lobe just below the diaphragm, near and along the left coronary ligament and the left triangular ligament. Moreover, the co-occurrence of liver ruptures located close to various ligaments (including the right triangular and the falciform ligament) with ruptures with other features, mainly those attributable to compression and crush injuries, was noteworthy. Indeed, the occurrence of such injuries during falls from heights and traffic accidents is not surprising.

All liver ruptures analysed were simultaneous ruptures of the capsule and parenchyma (rather than the capsule alone). They were divided into the following three main groups:

1. Ruptures located exclusively close to and parallel to the ligaments of the liver—a total of 13 cases, accounting for 21.31% (13/61) of all liver ruptures, 2 after falls from height and 11 after traffic accidents;
2. Concurrent ruptures located close to and parallel to the ligaments of the liver and liver ruptures with other features (stellate-type ruptures, crushing of the liver, linear lacerations unrelated to the course of the ligaments)—a total of 27 cases, accounting for 44.26% (27/61) of all liver ruptures, 12 after falls from height and 15 after traffic accidents;
3. Liver ruptures with other features (stellate-type ruptures, crushing of the liver, linear lacerations unrelated to the course of the ligaments), located away from the ligaments of the liver—a total of 21 cases, accounting for 34.42% (21/61) of all liver ruptures, 11 after falls from height and 10 after traffic accidents.

The final results for liver ruptures located at the ligaments of the liver were as follows:

1. Rupture on the diaphragmatic surface of the right lobe between both sectors, running parallel to the right triangular ligament: 17 cases (8 isolated, 9 coexisting with ruptures with other features).
2. Rupture on the diaphragmatic surface of the left lobe along and near the falciform ligament to the right (in segment IV) or to the left (in segment III) of it: 3 cases (1 isolated, 2 coexisting with fractures with other features).
3. Rupture on the diaphragmatic surface of the left lobe of the liver located just below the diaphragm, near and along the left coronary ligament and the left triangular ligament: 5 cases (1 isolated, 4 coexisting with ruptures with other features).
4. Simultaneous rupture on the diaphragmatic surface of the right lobe between both sectors, running parallel to the right triangular ligament, and rupture on the diaphragmatic surface of the left lobe along and near the falciform ligament to the right (in segment IV) or left (in segment III) of it: 6 cases (3 isolated, 3 coexisting with ruptures with other features).
5. Concomitant rupture on the diaphragmatic surface of the right lobe between both sectors, running parallel to the right triangular ligament, and rupture on the diaphragmatic surface of the left lobe of the liver located just below the diaphragm, close to and along the left coronary ligament and the left triangular ligament: 3 cases (all coexisting with ruptures with other features).
6. Rupture on the diaphragmatic surface of the left lobe along and near the falciform ligament to the right (in segment IV) or to the left (in segment III) of it and rupture on the diaphragmatic surface of the left lobe of the liver located just below the diaphragm, near and along the left coronary ligament and the left triangular ligament: 6 cases (all concurrent with ruptures with other features).
7. No liver ruptures located concomitantly in all three locations associated with the hepatic ligaments.

Among the total of 61 liver ruptures analysed, ruptures at the ligaments were found in 40 cases (14 in falls from height and 26 in traffic accidents), or 65.57% of cases. Among these 40 cases, a total of 55 liver ruptures were found located at the ligaments, divided as follows:

1. A total of 26 were on the diaphragmatic surface of the right lobe between both sectors, running parallel to the right triangular ligament;
2. A total of 15 were on the diaphragmatic surface of the left lobe along and near the falciform ligament to the right (in segment IV) or to the left (in segment III) of it;
3. A total of 14 were on the diaphragmatic surface of the left lobe of the liver located just below the diaphragm, near and along the left coronary ligament and the left triangular ligament.

The total number of liver ruptures located at the ligaments (55) was higher than the number of cadavers in which liver rupture was found at the ligaments (40) due to the fact that, in some cases (15), there were concomitant ruptures at two ligaments. All calculations were presented in an article detailing the issue analysed (9). Among other things, they showed a statistically significant incidence of liver rupture at the newly described site (i.e., within the upper part of the left lobe, in segment II, running along the left coronary ligament and the left triangular ligament).

## 4. Discussion

The results of the present study confirm the existence and highlight the importance of a specific liver injury—a transverse rupture located on the diaphragmatic surface of the left lobe of the liver in the subdiaphragmatic part, in segment II according to Couinaud—as a characteristic sequela of deceleration injuries. Although ruptures of the liver associated with forces pulling through its ligamentous apparatus are a well-known phenomenon [1,2,3,4], the results of this study indicate a relatively high incidence of damage in this little-known location, which, due to its site, shape, and course, may be overlooked during standard surgical inspection. The mechanism of the analysed rupture in segment II is directly related to the anatomy and biomechanics of the left liver.

The results of this study also represent the first broad and case-based description paying special attention to aspects related to the intraoperative confirmation or exclusion of the presence of a rupture of the lateral area of the left upper lobe of the liver, with a course transverse to the long axis of the body, along the diaphragm and the left coronary and triangular ligaments of the liver.

The left coronary ligament of the liver is formed by the separation of the falciform ligament into the right and left coronary ligaments. The role of the left coronary ligament is to attach the diaphragmatic portion of the liver to the diaphragm, and an extension of this ligament is the left triangular ligament [10,11,12]. The discussed rupture of the upper part of the left lobe of the liver, along the left coronary ligament and the left triangular ligament, in some cases coexisted with a rupture parallel to either the falciform ligament or the right triangular ligament. These two liver ruptures are attributed to the damaging action of these ligaments, i.e., the falciform ligament and the right triangular ligament, in the course of deceleration trauma [1,2,3,4]. This fact is evidence of the involvement of this mechanism in the formation also of a rupture in the upper part of the left lobe of the liver (in segment II according to Couinaud) running parallel to the diaphragm.

**Figure 1:**
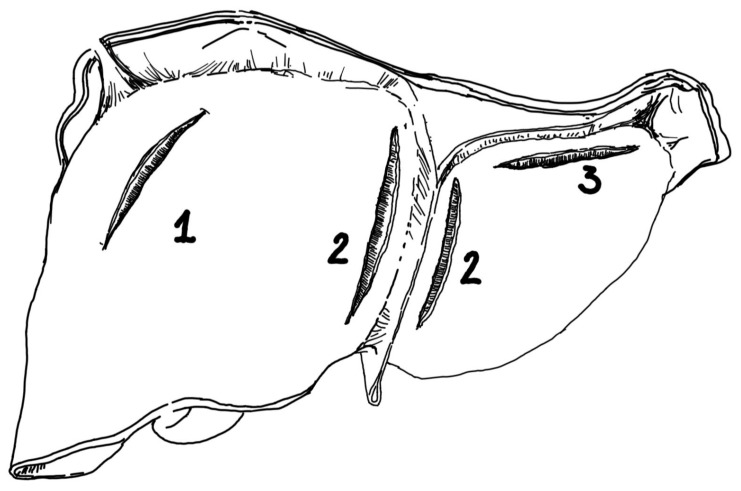
Ruptures of the liver following deceleration trauma, located close to the hepatic ligaments: 1—in the right lobe, parallel to the right triangular ligament; 2—in the left lobe, in close proximity to and along the falciform ligament (to the right or left of the ligament); 3—in the left lobe, parallel to the diaphragm, close to and along the left coronary ligament and the left triangular ligament. Source: authors’ own work.

All deceleration ruptures of the liver located in characteristic locations, near and along the ligaments, such as right triangular, falciform, left coronary, and left triangular ligaments, are shown in Figure 1.

For every patient operated on for both blunt and penetrating trauma, it is mandatory to check all abdominal structures (not only organs, but also blood vessels and ligaments) for possible post-traumatic injuries. This is because it is possible for different injuries to occur concomitantly [13,14].

Liver ruptures located close to the ligaments were described long ago, although either without indicating a link between their origin and deceleration trauma or only indicating the ligaments as a contributing factor, but without a clear link to deceleration [8,15,16]. It is also worth noting that ruptures of the liver capsule and parenchyma can be caused by the so-called “bursting effect” in the course of liver compression, comparable to rupturing when crushing a paper bag filled with air [8]. Impact to the anterior surface of the liver causes frontal crush injury and leads to the damage of central segments IV, V, and VIII according to Couinaud [1]. Liver ruptures caused by compression of the ribs against the liver can have a characteristic appearance described as a “bear claw” [17,18]. Conte et al. experimentally demonstrated the following five characteristic patterns of compressive liver ruptures:

1. Laceration of the diaphragmatic face of the right lobe next to the bare area;
2. Laceration of the right anterior lateral face with extension to the posterior face;
3. Laceration of the lower part of the right lobe affecting (in some cases) more than half of the lobe;
4. Laceration initiated next to the vena cava;
5. Crush of the parenchyma [19].

Ruptures such as those indicated above were found in the study and described as ‘liver ruptures with other features’.

The analysed liver injury, i.e., a rupture parallel to the left coronary ligament and its extension, the left triangular ligament, located within segment II of the liver according to Couinaud, was very rarely described in the literature, or its presence could at best only be considered on the basis of suggested general mechanisms of trauma leading to liver ruptures. In the early 1970s, Hardy, presenting injury patterns on the anterior surface of the liver, admittedly identified, among other things, a “transverse rupture” of the left lobe of the liver, located high up and running parallel to the diaphragm [8]. However, he did not consider it to be a rupture caused by ligamentous action in the course of shearing forces. Walt pointed out that the liver is a heavy mobile organ, suspended from the diaphragm by the coronary and triangular ligaments and maintaining vital connections through the portal triad and the delicate hepatic veins. He believed that sudden shearing forces directed at the liver could cause tears of the parenchyma near the attachment of the suspensory ligaments. However, he did not specifically indicate the location of these injuries [16]. In contrast, in the aforementioned article, Hardy pointed out that sudden shearing forces could result simultaneously in tears close to the ligaments’ attachments and at the same time indicated that longitudinal, rather than transverse, tears of the anterior aspect of the liver, with no posterior–superior segmental shattering, are most likely to occur in this manner [8].

A rupture located in the upper part of the left lobe of the liver, in segment II, running along the left coronary ligament and the left triangular ligament, may be overlooked during inspection of the abdominal organs during a laparotomy performed after blunt abdominal trauma. Overlooking this injury may be due to the following reasons:

- Location high within the diaphragmatic surface of the liver and just below the diaphragm;
- It is transverse (rather than longitudinal) to the long axis of the body course of the rupture fissure, additionally parallel to and in close proximity to the diaphragm;
- Relatively few descriptions of such a rupture in the literature (perhaps due to the infrequent finding of this liver injuries) and thus also little awareness of the existence of such a hepatic rupture in general.

The clinical consequences of overlooking a subhepatic rupture of segment II of the left lobe of the liver may mainly include prolonged bleeding with haematoma formation and bile leakage with biloma formation (depending on the depth of the liver parenchymal injury).

The first factor that has a significant impact on the intraoperative assessment of liver injury is the type of incision by which the abdominal cavity is to be opened. If the preoperative diagnostic imaging studies show the presence of liver injury, then it is likely that the abdominal cavity will be opened from a bilateral subcostal incision. If, on the other hand, imaging studies show the presence of multiple injuries or are not performed due to the patient’s severe condition requiring urgent laparotomy, then the situation will be different. Most likely, a median incision will be chosen, and the initial intraoperative assessment will be carried out from this access. Subsequently, if necessary, the median incision may be widened with a subcostal cut (right or bilateral). Obviously, assessment of the liver may be easier and performed more quickly if the abdominal cavity is reached through an incision originally guided parallel to the rib arches. 

**Figure 2:**
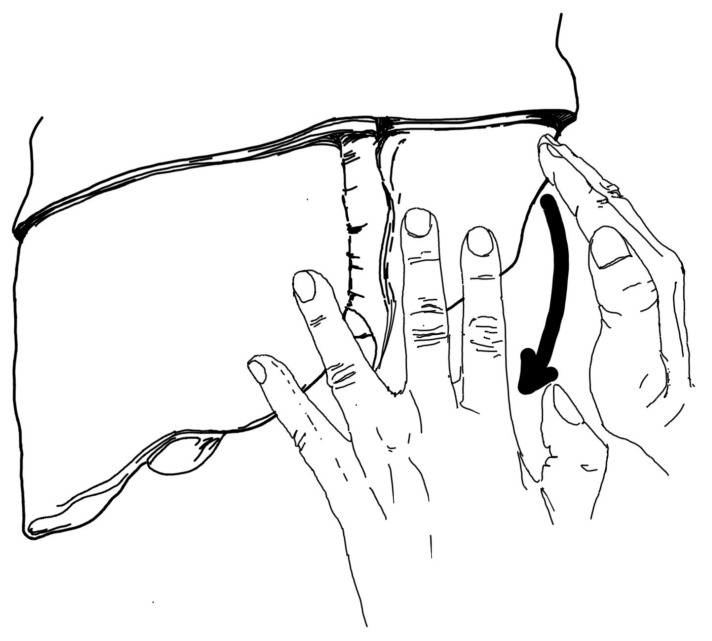
Carefully pulling the liver backwards and downwards with the hands. Source: authors’ own work.

**Figure 3:**
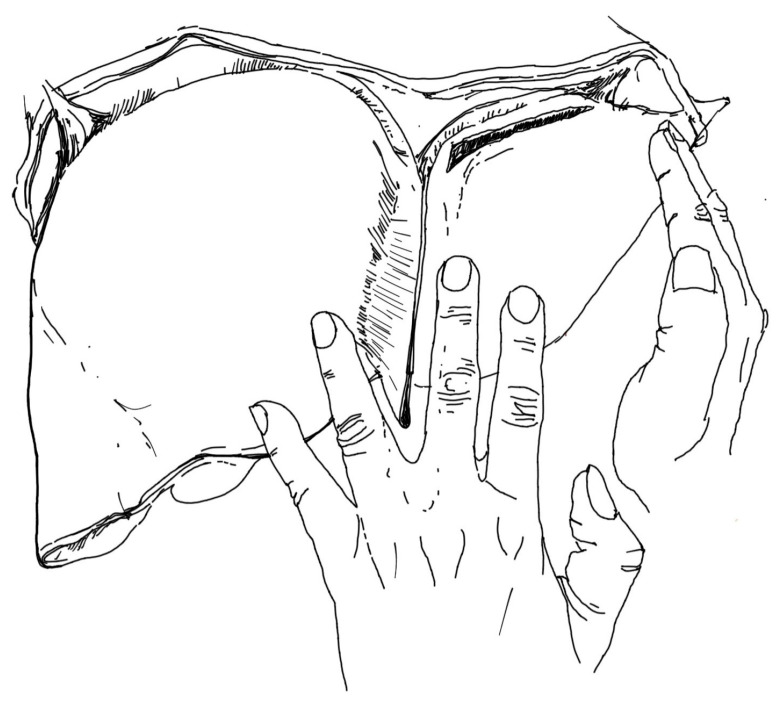
Visualisation of the upper-lateral area of the diaphragmatic surface of the left hepatic lobe with a rupture parallel to the left coronary ligament and the left triangular ligament of the liver. Source: authors’ own work.

The proposed method of intraoperative assessment of the lateral part of the left lobe of the liver consists of the following steps:

1. Position of the hands on the right and left lobes of the liver in such a way that the right hand holds only the lateral area of the left lobe of the liver and presses it against the rest of the organ. In contrast, the left hand rests on the diaphragmatic surface of the liver in such a way that fingers II-III are to the left (on segment III) of the falciform ligament and IV-V are to the right of this ligament (on segment IV).
2. Gently pulling the liver with the hands in a posterior-basal direction, not just inferiorly (Figure 2). Too much downward pulling of the liver could, in fact, enlarge the tear of the liver parenchyma, having a transverse course. Pressing the left lobe of the liver with the right hand is intended to prevent this from happening, also with regard to ruptures located in other areas of the organ. In this way, the subdiaphragmatic area of segment II of the left liver lobe should be visualised (Figure 3).
3. Transection of the falciform ligament if the liver could not be moved posteriorly and downwards to the extent that the entire area of the diaphragmatic surface of the left lobe could be visualised, especially the part located just below the diaphragm.

Further intraoperative management depends on the condition found during visual and palpation assessment. If the liver needs to be mobilised, then the falciform ligament should be cut in the usual way if this has not been done previously.

The proposed method of assessing the subhepatic area of the left lobe of the liver within segment II may also be helpful in detecting possible injuries involving the diaphragm. Indeed, cases of liver rupture accompanied by diaphragmatic rupture have been described [20,21]. In addition, it will then be possible to confirm or exclude the presence of type B juxtahepatic venous injury, i.e., damage to the veins in the extrahepatic area, accompanied by rupture of the hepatic suspensory ligaments, the diaphragm, or the ligaments and diaphragm concomitantly [12]. To inspect this region, however, it is necessary to transect the coronary and triangular ligaments of the left lobe of the liver.

It is also worth noting another aspect arising from the study, related to the coexistence of ruptures of the capsule and liver parenchyma at more than one ligament. As already mentioned, a rupture along the left coronary ligament and the left triangular ligament, for example, can occur together with a rupture parallel to the falciform ligament or the right triangular ligament, i.e., liver injuries attributed to the damaging action of these ligaments in the course of deceleration trauma. It is not difficult to spot a rupture of the liver at the falciform ligament or the right triangular ligament, if only because they run longitudinally to the long axis of the body (or close to it). On the other hand, the finding of even one of these ruptures should oblige the surgeon to inspect the liver very carefully in the vicinity of its other ligaments, including the left coronary and left triangular ligaments, due to the reasonable suspicion that the patient has suffered a deceleration trauma. However, inspection of this area should also occur if ruptures at other ligaments are not found.

The results of this study may fill an existing gap in the literature regarding this specific pattern of liver injury, but more importantly, they can provide surgeons with practical guidelines that may contribute to increasing the detection of these ruptures. By gaining a better understanding of the pathomechanics and morphology and by implementing targeted intraoperative assessment, it is possible to minimize the risk of unrecognized liver injury and consequently improve the outcome of patients following severe deceleration injuries.

The location of the rupture in question in the upper part of the liver and parallel to the diaphragm, just posterior to it, carries an aspect that can be exploited in the final phase of the surgical operation. Well, the diaphragm adjacent to the liver can press haemostatic materials against it. Studies demonstrate the beneficial effects of these materials in reducing local bleeding from the liver [22,23,24].

The results of the present study indicate the need for further research in this area. Prospective clinical studies assessing the actual incidence of segment II left lobe ruptures in patients operated on for blunt abdominal trauma and the effectiveness of the proposed intraoperative inspection methodology would be valuable. The study carried out was of fatal cases and does not provide direct information on the course of treatment and prognosis in patients who survived similar injuries.

## 5. Conclusions

1. A transverse rupture of the left lobe of the liver in segment II according to Couinaud, in its subdiaphragmatic area, arises as a result of the “pulling” forces of the ligaments during deceleration trauma and belongs to the group of characteristic ruptures of this organ arising in such circumstances and located close to the ligaments of the liver.
2. The presence of a rupture of segment II of the left lobe of the liver running parallel to and just below the diaphragm should always be considered in the event of a blunt abdominal trauma, especially one in which deceleration was highly likely to be involved (e.g., when a rupture of the liver at its other ligament is present). Therefore, the subdiaphragmatic area of the left lateral lobe of the liver should be inspected intraoperatively.
3. The presence of a rupture of the liver at one of its ligaments should raise the suspicion of a concomitant rupture at one of the other ligaments.

## Data Availability

The original contributions presented in this study are included in the article. Further inquiries can be directed to the corresponding authors.
